# Discovery of a new hypotrich ciliate from petroleum contaminated soil

**DOI:** 10.1371/journal.pone.0178657

**Published:** 2017-06-01

**Authors:** Santosh Kumar, Daizy Bharti, Shahed Uddin Ahmed Shazib, Mann Kyoon Shin

**Affiliations:** Department of Biological Sciences, College of Natural Sciences, University of Ulsan, Ulsan, South Korea; University of Ostrava, CZECH REPUBLIC

## Abstract

Pollution after oil spill represents extreme habitat for survival and is a major concern for loss of species diversity in the affected area. In this study, we investigated soil samples collected from a petrochemical industry, Ulsan, South Korea. The soil was in the phase of recovery from the contamination of crude oil spill. Detailed investigation, based on morphology, ontogenesis, and molecular phylogenetic methods, resulted in discovery of a novel hypotrich ciliate, i.e., *Metasterkiella koreana* n. gen., n. sp., which is morphologically characterized by a semirigid body, undulating membranes in *Oxytricha* pattern, 18 frontal-ventral-transverse cirri with cirrus V/3 placed posteriorly, one right and one left row of marginal cirri, four dorsal kineties, two dorsomarginal rows, and caudal cirri at the end of dorsal kineties 1, 2, and 4. Interestingly, during ontogenesis, formation of three common anlagen for the proter and the opisthe and involvement of cirrus V/3 in anlagen formation was observed. The dorsal ontogenesis was typical of oxytrichids, i.e., simple fragmentation of dorsal kinety 3 and formation of dorsomarginal rows close to the right marginal row. The new species was found to be similar with *Sterkiella subtropica*, except for some minor differences in morphometry, and at gene level with only one base pair difference. In phylogenetic analyses, based on SSU rRNA gene sequence, *M*. *koreana* cluster in a clade away from *Sterkiella* species, which could be explained by the differences in the morphogenetic pattern between these two genera. It is proposed that *S*. *subtropica* probably belongs to *Metasterkiella*; however, we do not perform changes and wait for the reinvestigation of its morphogenetic pattern.

## Introduction

It is not uncommon to find morphological variability within and between populations in ciliated protozoa [1–5]. Most of this variability is the result of laboratory culture conditions and stressful environmental conditions. Recent studies have shown that careful observations have resulted in recognition of cryptic ciliate species [3–5]. Indeed, crypticity is often predicted in molecular phylogenetic analyses and can be explained by insufficient acquisition and/or interpretation of the data. One example for this is represented by cyst species, a very recent concept in ciliates, but it can be estimated that it will significantly increase the number of ciliate species [3, 6, 7].

However, when ontogenesis within the genus is compared, most congeners have a rather similar mode with some minor variations [8]. In the present study, we investigated, in detail, the ontogenetic patterns of a novel hypotrich ciliate, i.e., *Metasterkiella koreana* n. gen., n. sp., which resembled morphologically very well with one *Sterkiella* species. Nonetheless, we observed a rather different mode of division which differs significantly when compared with the detailed ontogenetic data available for the genus *Sterkiella* [1, 2]. Thus, we established a novel genus i.e., *Metasterkiella* for the novel species in accordance with the molecular analyses, which showed that the genus *Sterkiella* is a polyphyletic assemblage. We feel that such variable characters still exist within the genus and detailed investigation of which (i.e., morphology, ontogenesis, cyst structure) will help in differentiating the species often assigned to collective groups, e.g., *Sterkiella histriomuscorum* complex. Another example of morphological differentiation was presented in Kumar et al. [9] who described a novel *Sterkiella* species based on difference from other species by having constantly four (vs. five) transverse cirri. Similarly, Foissner [3] identified a novel species, i.e., *Fragmospina depressa*, which resembled with *S*. *histriomuscorum* but had undulating membranes close (vs. distant) to adoral zone of membranelles and resting cyst spinous (vs. wrinkled). All these examples suggest a fast evolution of the *Sterkiella*-like hypotrichs possibly originating from phylogenetically distant species.

## Materials and methods

### Description of the sampling site and sample processing

Soil samples were collected from the spilled oil treatment facility, Onsan, Ulsan, South Korea (35° 24' 56"N; 129° 20' 38"E) on April 8^th^, 2016, under control of the Safety Manager with prior permission from the company. Ciliates were reactivated from resting cysts from one-month-dried soil samples (approximately 300 g) by employing the non-flooded Petri dish method [10]. A clonal culture of *M*. *koreana* was established as described in Kumar et al. [9], i.e., using Pringsheim’s medium for culturing and the green alga *Chlorogonium elongatum* as food organism. Live observations were made using a microscope with bright-field and differential interference contrast illuminations at a magnification of 100–1000×. The protargol staining method described by Kamra and Sapra [11] was used with some modification to reveal the ciliature. Measurements of impregnated specimens were performed at a magnification of 1000× using an ocular micrometer. A Zeiss microscope camera was employed for photomicrography. The illustration of the live specimen was prepared using free-hand sketches, while those of impregnated specimens were made with a drawing device. Terminology is according to Berger [1] and Wallengren [12].

### DNA extraction, PCR amplification, and sequencing

Five cells were collected from a clonal culture with the help of glass micropipettes and washed three times with autoclaved distilled water (same culture was used for live observation and protargol staining to study morphology and ontogenesis). Genomic DNA was extracted using the RED Extract-N-Amp Tissue PCR Kit (Sigma, St. Louis, MO), following the manufacturer’s instruction except for the reduction of each reaction volume to one-tenth [13]. Extracted DNA (1 μl) was dispensed into a PCR tube containing 22 μl of autoclaved distilled water, and amplifications were carried out using the TaKaRa ExTaq DNA polymerase Kit (TaKaRa Bio-medicals, Otsu, Japan) in a total volume of 30 μl with the universal eukaryotic primers Euk A (FW 5'-AAC CTG GTT GAT CCT GCC AG-3') and Euk B (RV 5'-CAC TTG GAC GTC TTC CTA GT-3') [14]. The PCR program for SSU rRNA gene amplification included an initial denaturation at 94°C for 3 min, followed by 35 cycles of 94°C for 1 min, 56°C for 45 s and 72°C for 80 s, with a final extension step at 72°C for 10 min. After confirmation of the appropriate size in 1.2% agarose gel, the purified PCR products were directly sequenced on both strands on an ABI 3730 automatic sequencer (Cosmo genetech., Seoul, Korea).

### Phylogenetic analyses

For phylogenetic analyses, the SSU rRNA gene sequence of *M*. *koreana* was aligned with 54 SSU rRNA gene sequences of hypotrich ciliates from GenBank using the MAFFT software v. 7.047 (choosing the iterative refinement methods Q-INS-I that considers the secondary structure of the SSU rRNA molecules) [15].

Ambiguously aligned regions were identified and excluded from the phylogenetic analyses with GBlocks v.0.91b [16] using parameters optimized for rRNA alignments, leaving 1,514 unambiguous positions. A Bayesian inference (BI) analysis was performed using MrBayes v.3.2.1 [17] and the TIM2+I+G model, as selected by the jModel test v.2.1.3 software [18] under the *Akaike Information Criterion corrected* (AICc). Markov chain Monte Carlo (MCMC) simulations were run, with two sets of four chains using the default settings, for 1,000,000 generations with trees sampled every 100 generations and discarding the first 25% of the sampled trees as burn-in. The remaining trees were used to generate a consensus tree and to calculate the posterior probabilities (PP) of all branches using the majority-rule consensus approach. Maximum likelihood (ML) analyses were carried out using RAxML-HPC2 v. 8.0.24 [19] on the CIPRES Science Gateway [20] with bootstrapping of 1000 replicates. Phylogenetic trees were visualized using the free software package FigTree v. 1.4 by A. Rambaut at http://tree.bio.ed.ac.uk/software/figtree/. To assess the probability of the monophyletic relationships between *Metasterkiella koreana* and the genus *Sterkiella*, the approximately unbiased (AU) test was performed in CONSEL v. 0.1j [21, 22]. Steps were as described in Shazib et al. [23]. Two constrained ML analyses were carried out, i.e., (i) monophyly of *M*. *koreana* and *Sterkiella cavicola*, and (ii) monophyly of *M*. *koreana* and all other *Sterkiella* species.

### Data availability

The newly obtained SSU rRNA gene sequence of *M*. *koreana* is available from the GenBank/EMBL databases (accession number: KY448243). Slides with fixed specimens are available from the National Institute of Biological Resources (NIBR), Incheon, Korea with registration numbers NIBRPR0000107886, NIBRPR0000107887, and NIBRPR0000107888. The slides contain many specimens, with relevant cells marked by a black ink circle on the cover glass.

### Nomenclatural acts

The electronic edition of this article conforms to the requirements of the amended International Code of Zoological Nomenclature, and hence the new names contained herein are available under that Code from the electronic edition of this article. This published work and the nomenclatural acts it contains have been registered in ZooBank, the online registration system for the ICZN. The ZooBank LSIDs (Life Science Identifiers) can be resolved and the associated information viewed through any standard web browser by appending the LSID to the prefix “http://zoobank.org/”. The LSID for this publication is: urn:lsid:zoobank.org:pub:ADCE754A-CD51-4D72-AF73-B31813D36C5D. The electronic edition of this work was published in a journal with an ISSN, and has been archived and is available from the following digital repositories: PubMed Central, LOCKSS.

## Results

### Description of *Metasterkiella koreana* n. gen. n. sp.

**Fig 1:**
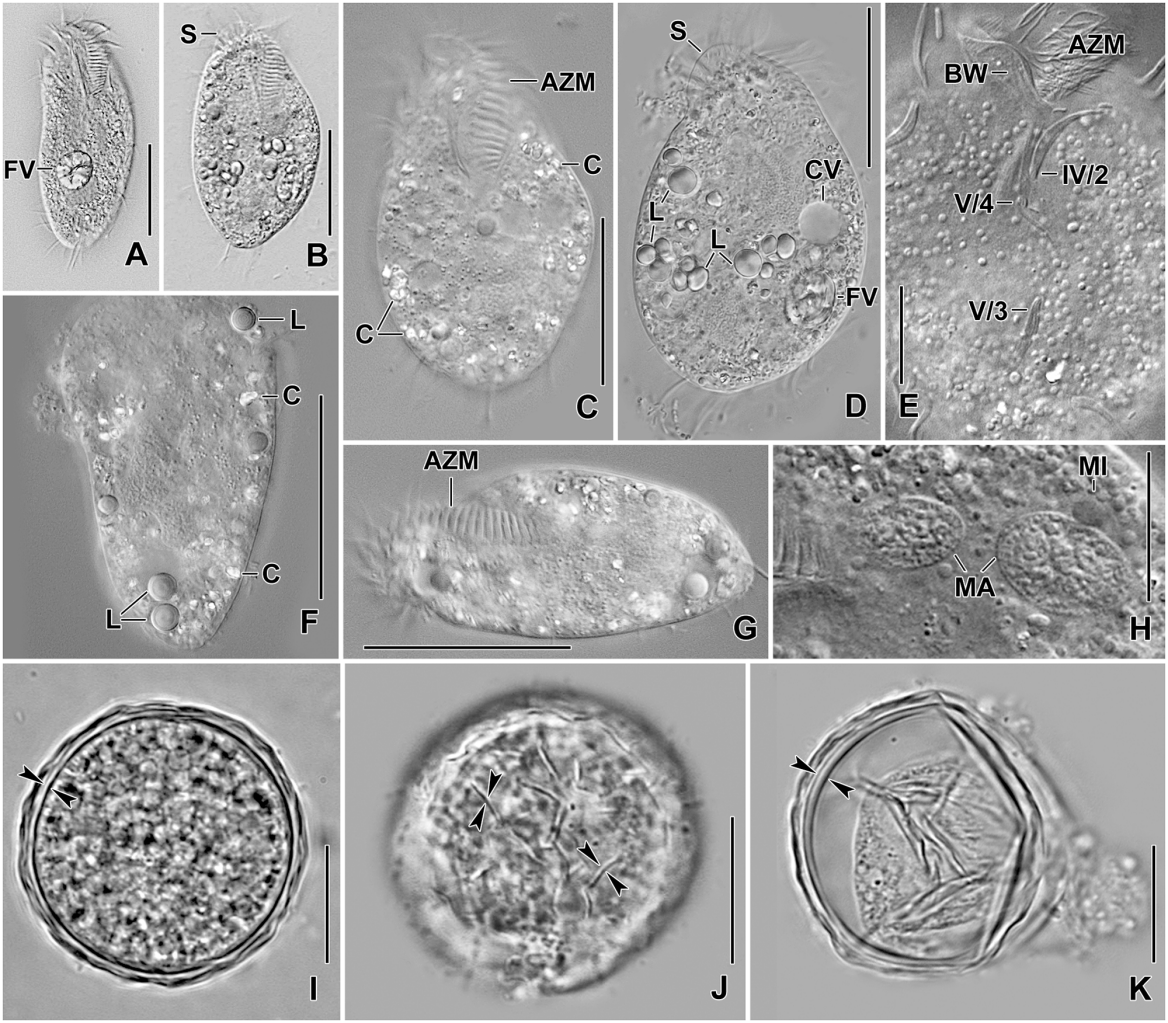
Photomicrographs of *Metasterkiella koreana* from life. (**A, B**) Specimens, showing body shape. (**C, D, G**) Slightly compressed specimens due to cover slip pressure, showing cytoplasmic crystals and lipid droplets. **(E)** Section, showing the buccal wall and postoral ventral cirri. **(F)** A squeezed specimen, showing the lipid droplets and the cytoplasmic crystals mainly around the margin of the cell. **(H)** Nuclear apparatus in ventral view. (**I–K**) Resting cyst. Optical section (I), showing the cyst wall (opposed arrowheads). Surface view (J), showing the wrinkled hyaline ridges (opposed arrowheads). Squeezed cyst (K), showing cyst wall (opposed arrowheads). AZM, adoral zone of membranelles; BW, buccal wall; C, crystals; CV, contractile vacuole; FV, food vacuoles; L, lipid droplets; MA, macronuclear nodules; MI, micronuclei; S, scutum; IV/2, V/4, V/3, postoral ventral cirri. Scale bars = 15 μm (E, H–K) and 40 μm (A–D, F, G).

**Fig 2:**
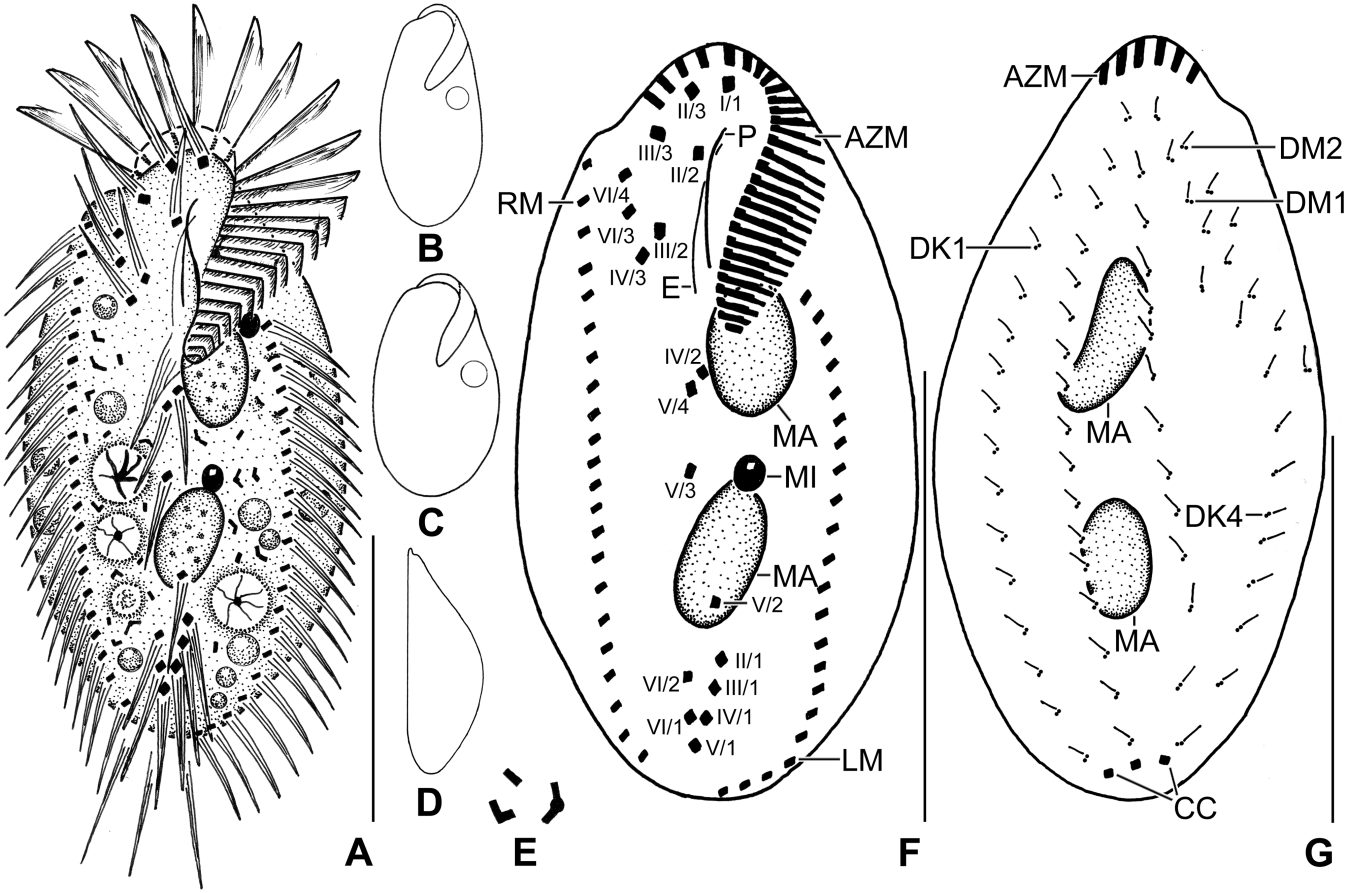
Line diagrams of *Metasterkiella koreana* from life (A–E) and after protargol impregnation (F, G). **(A)** A representative cell with a length of 85 μm. (**B, C**) Body shape of partially starved (B) and well fed (C) specimens. **(D)** Dorsolateral view. **(E)** Cytoplasmic crystals. (**F, G**) Ventral view of the holotype (F) and dorsal view of a paratype specimen (G), showing the ciliature and the nuclear apparatus. AZM, adoral zone of membranelles; CC, caudal cirri; DK1,4, dorsal kineties; DM1,2, dorsomarginal kineties; E, endoral membrane; LM, left marginal row; MA, macronuclear nodules; MI, micronuclei; P, paroral membrane; RM, right marginal row; I/1, II/3, III/3, frontal cirri; II/2, buccal cirrus; III/2, IV/3, VI/3, VI/4, fronto-ventral cirri; IV/2, V/4, V/3, postoral ventral cirri; V/2, VI/2, pretransverse ventral cirri; II/1, III/1, IV/1, V/1, VI/1, transverse cirri. Scale bars = 40 μm.

**Fig 3:**
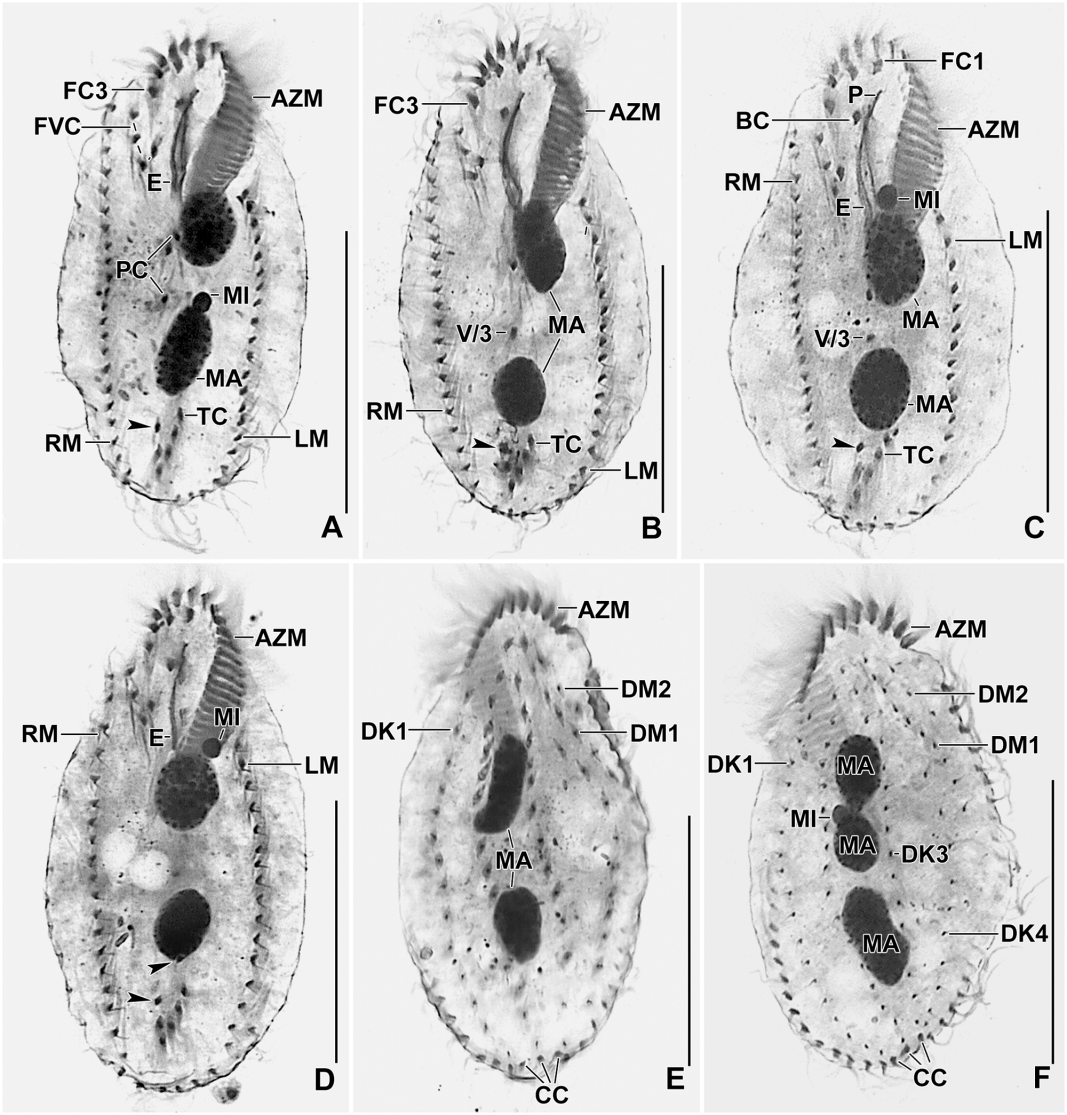
Photomicrographs of *Metasterkiella koreana* after protargol impregnation. **(A)** Ventral view of the holotype specimen. **(B–F)** Paratype specimens, showing body shape, nuclear apparatus, and ciliature of the ventral (B–D) and dorsal surface (E, F). Rarely a specimen (F) with three macronuclear nodules occurs. Arrowheads in (A–D) point to the pretransverse ventral cirri. AZM, adoral zone of membranelles; BC, buccal cirrus; CC, caudal cirri; DK1,3,4, dorsal kineties; DM1,2, dorsomarginal kineties; E, endoral membrane; FC (1, 3), frontal cirri; FVC, frontoventral cirri; LM, left marginal row; MA, macronuclear nodules; MI, micronuclei; P, paroral membrane; PC, postoral cirri; RM, right marginal row; TC, transverse cirri; V/3, postoral ventral cirrus. Scale bars = 40 μm.

Size in vivo 75–100 × 40–75 μm, usually about 85 × 50 μm, as calculated from some in vivo (n = 5) measurements and morphometric data in Table 1, assuming 15% preparation shrinkage [5]. Body outline oval, elliptical to broadly elliptical, both ends broadly rounded; dorso-ventrally flattened about 3:2 (Figs 1A–1D, 1G, 2A–2D, 2F, 2G and 3A–3F; Table 1). Nuclear apparatus in or slightly left of midline, composed of two macronuclear nodules and one or two micronuclei (Figs 1H, 2A, 2F and 3A–3F; Table 1). Macronuclear nodules globular to broadly ellipsoidal, anteriormost nodule on average 13 × 9 μm in protargol preparations; contain many small nucleoli, 1–3 μm across. Micronuclei usually attached to or near to macronuclear nodules, globular, on average 3.0 μm across in protargol preparations (Figs 1H, 2A, 2F and 3A–3F; Table 1). Contractile vacuole left of body’s midline, near cell margin (Figs 1D and 2A). Cortex semirigid; cortical granules absent. Cytoplasm colorless, filled with few to many crystals of about 2–4 μm in size and some fat droplets about 3–6 μm in diameter (Figs 1D–1F and 2A and 2E). Feeds on bacteria and small flagellates, and starch grains from squashed wheat kernels in non-flooded Petri dish culture (Figs 1A, 1B, 1D and 2A). Swims and creeps rather rapidly.

**Table 1:** Morphometric data on *Metasterkiella koreana* n. gen., n. sp.

Characteristic^a^	Mean	M	SD	SE	CV	Min	Max	n
Body, length	73.4	74.0	5.1	1.0	6.9	65.0	83.0	25
Body, width	41.8	41.0	6.7	1.3	16.0	34.0	66.0	25
Body length:width, ratio	1.8	1.8	0.2	0.0	11.1	1.1	2.1	25
Anterior body end to proximal end of adoral zone, distance	28.4	29.0	2.9	0.6	10.3	23.0	34.0	25
Body length:AZM length, ratio	2.6	2.6	0.2	0.0	8.9	2.2	3.3	25
Anterior body end to proximal end of adoral zone, % of body length	38.7	38.7	3.2	0.6	8.3	29.9	45.5	25
Adoral membranelles, number	24.0	24.0	1.7	0.4	7.2	21.0	28.0	23
Adoral membranelles, width of largest base	6.7	7.0	0.7	0.1	9.7	6.0	8.0	19
Anterior body end to paroral membrane, distance	8.8	9.0	1.1	0.2	12.1	7.0	11.0	19
Anterior body end to endoral membrane, distance	11.0	11.0	1.0	0.2	9.1	9.0	13.0	17
Anterior body end to anterior macronuclear nodule, distance	23.9	24.0	2.3	0.5	9.8	20.0	28.0	19
Posterior body end to posterior macronuclear nodule, distance	16.2	16.0	1.7	0.4	10.4	12.0	20.0	19
Macronuclear figure, length	33.8	34.0	3.9	0.9	11.5	27.0	41.0	19
Distance between macronuclear nodules	8.0	9.0	2.3	0.5	29.2	5.0	12.0	19
Anterior macronuclear nodule, length	13.2	13.0	2.3	0.5	17.4	10.0	17.0	19
Anterior macronuclear nodule, width	8.9	9.0	1.1	0.2	12.1	6.0	11.0	19
Posterior macronuclear nodule, length	12.6	13.0	1.9	0.4	14.8	10.0	17.0	19
Posterior macronuclear nodule, width	8.0	8.0	1.8	0.4	22.4	5.0	11.0	19
Macronuclear nodules, number	2.0	2.0	0.0	0.0	0.0	2.0	2.0	25
Anterior body end to anterior micronucleus, distance	23.6	23.0	2.3	0.6	9.8	20.0	28.0	15
Anterior micronucleus, length	3.1	3.0	0.4	0.1	12.1	2.5	4.0	17
Anterior micronucleus, width	2.6	2.5	0.4	0.1	15.3	2.0	3.0	17
Micronuclei, number	1.8	2.0	0.4	0.1	21.5	1.0	2.0	17
Anterior body end to right marginal row, distance	12.8	12.0	2.1	0.5	16.8	10.0	17.0	19
Posterior body end to right marginal row, distance	4.7	5.0	1.4	0.4	29.3	2.0	7.0	15
Right marginal row, number of cirri	21.1	21.0	1.1	0.3	5.2	19.0	23.0	19
Anterior body end to left marginal row, distance	24.7	25.0	1.7	0.4	7.0	22.0	28.0	19
Posterior body end to left marginal row, distance	1.0	1.0	0.3	0.1	32.7	0.5	2.0	15
Left marginal row, number of cirri	20.1	20.0	1.1	0.3	5.5	18.0	22.0	19
Gap between last cirri of marginal rows	8.4	8.0	1.3	0.3	15.5	6.0	10.0	15
Frontal cirri, number	3.0	3.0	0.0	0.0	0.0	3.0	3.0	19
Anterior body end to buccal cirrus, distance	11.2	11.0	1.4	0.3	12.8	8.0	14.0	19
Buccal cirrus, number	1.0	1.0	0.0	0.0	0.0	1.0	1.0	19
Anterior body end to posteriormost frontoventral cirrus, distance	20.8	21.0	2.1	0.5	10.3	18.0	25.0	19
Frontoventral cirri, number	4.0	4.0	0.0	0.0	0.0	4.0	4.0	19
Anterior body end to posteriormost postoral cirrus, distance	42.9	44.0	3.9	0.9	9.1	37.0	50.0	19
Distance between cirrus V/3 and V/4	7.5	7.0	1.9	0.5	26.0	5.0	13.0	17
Postoral cirri, number	3.0	3.0	0.0	0.0	0.0	3.0	3.0	19
Pretransverse cirri, number	2.0	2.0	0.0	0.0	0.0	2.0	2.0	19
Posterior body end to rear transverse cirrus, distance	5.3	5.5	1.6	0.4	30.7	3.0	8.0	16
Transverse cirri, number	5.0	5.0	0.0	0.0	0.0	5.0	5.0	19
Dorsal kineties, number	6.0	6.0	0.0	0.0	0.0	6.0	6.0	15
Anterior body end to dorsal kinety 1, distance	15.3	16.0	3.1	0.8	20.5	9.0	19.0	15
Dorsal kinety 1, number of bristles	15.7	16.0	1.7	0.4	10.6	14.0	20.0	15
Anterior body end to dorsal kinety 2, distance	8.9	9.0	1.2	0.3	14.1	7.0	11.0	15
Dorsal kinety 2, number of bristles	15.9	16.0	1.2	0.3	7.7	14.0	18.0	15
Anterior body end to dorsal kinety 3, distance	12.8	13.0	2.3	0.6	18.0	8.0	15.0	15
Dorsal kinety 3, number of bristles	13.1	13.0	1.3	0.3	10.2	11.0	15.0	15
Anterior body end to dorsal kinety 4, distance	23.3	23.0	2.8	0.7	12.1	19.0	30.0	15
Dorsal kinety 4, number of bristles	10.1	10.0	1.0	0.3	9.8	8.0	11.0	15
Dorsomarginal row 1, number of bristles	7.7	7.0	1.0	0.3	13.4	7.0	10.0	15
Dorsomarginal row 2, number of bristles	3.0	3.0	0.4	0.1	12.6	2.0	4.0	15
Caudal cirri, number	2.9	3.0	0.4	0.1	12.3	2.0	3.0	15

^a^ Data based on mounted, protargol-impregnated, and randomly selected specimens from a clonal culture fed with *Chlorogonium elongatum*. Measurements in μm. CV—coefficient of variation in %, M—median, Max—maximum, Mean—arithmetic mean, Min—minimum, n—number of individuals investigated, SD—standard deviation, SE—standard error of arithmetic mean.

Cirral pattern and number of cirri rather constant. Invariably, 18 fronto-ventral-transverse cirri (Figs 2A, 2F and 3A–3D; Table 1). Frontal cirri slightly enlarged, in vivo about 15 μm long, right cirrus near distal end of adoral zone, middle cirrus anterior of buccal cirrus, left cirrus anterior of distal end of undulating membranes. One slightly thickened buccal cirrus about 11 μm from anterior body end. Four frontoventral cirri, arranged in opposed J-shaped pattern (Figs 2A, 2F and 3A–3D; Table 1). Three postoral and invariably two obliquely arranged pretransverse cirri. Five transverse cirri arranged in a hooked pattern, in vivo about 15 μm long, base of rearmost cirrus about 5 μm distant from posterior body end (Figs 1E, 2A, 2F and 3A–3C; Table 1). Marginal cirri about 12 μm long in protargol preparations. Left row reaches up to the posterior body end following the margin, composed of an average of 20 cirri; right row about 5 μm distant from posterior body end, composed of an average of 21 cirri (Figs 2A, 2F and 3A–3D; Table 1).

Invariably six dorsal kineties with bristles about 2–3 μm long in protargol preparations. Kineties 1–3 roughly bipolar; kinety 4 distinctly shortened anteriorly, commences at about 32% of body length; kineties 5 and 6 shortened posteriorly (Figs 2G and 3E and 3F; Table 1). Three narrowly spaced caudal cirri, about 20 μm long in vivo, one each at posterior ends of dorsal kineties 1, 2, and 4 (Figs 2G and 3E and 3F; Table 1).

Adoral zone extends about 39% of body length, on average composed of 24 membranelles with about 17 μm long cilia in vivo, bases of largest membranelles about 7 μm wide in protargol preparations (Figs 1C, 1G, 2A, 2F and 3A–3D; Table 1). Buccal cavity narrow, lip prominent about 5 μm wide in vivo (Figs 1E and 2A). Undulating membranes in body’s midline, slightly curved, intersect optically near buccal cirrus. Paroral commences about 9 μm from anterior body end; endoral commences about 2 μm posterior to distal end of paroral (Figs 2A, 2F and 3A–3D; Table 1).

### Resting cyst

Two-week-old resting cysts about 35 μm across in vivo; cyst surface with hyaline ridges, about 2.0 μm high (Fig 1I–1K). Cyst wall 1.0–1.5 μm thick. Cyst content composed of many lipid droplets 1.5–3.0 μm across in vivo (Fig 1I–1K).

### Notes on ontogenesis

**Fig 4:**
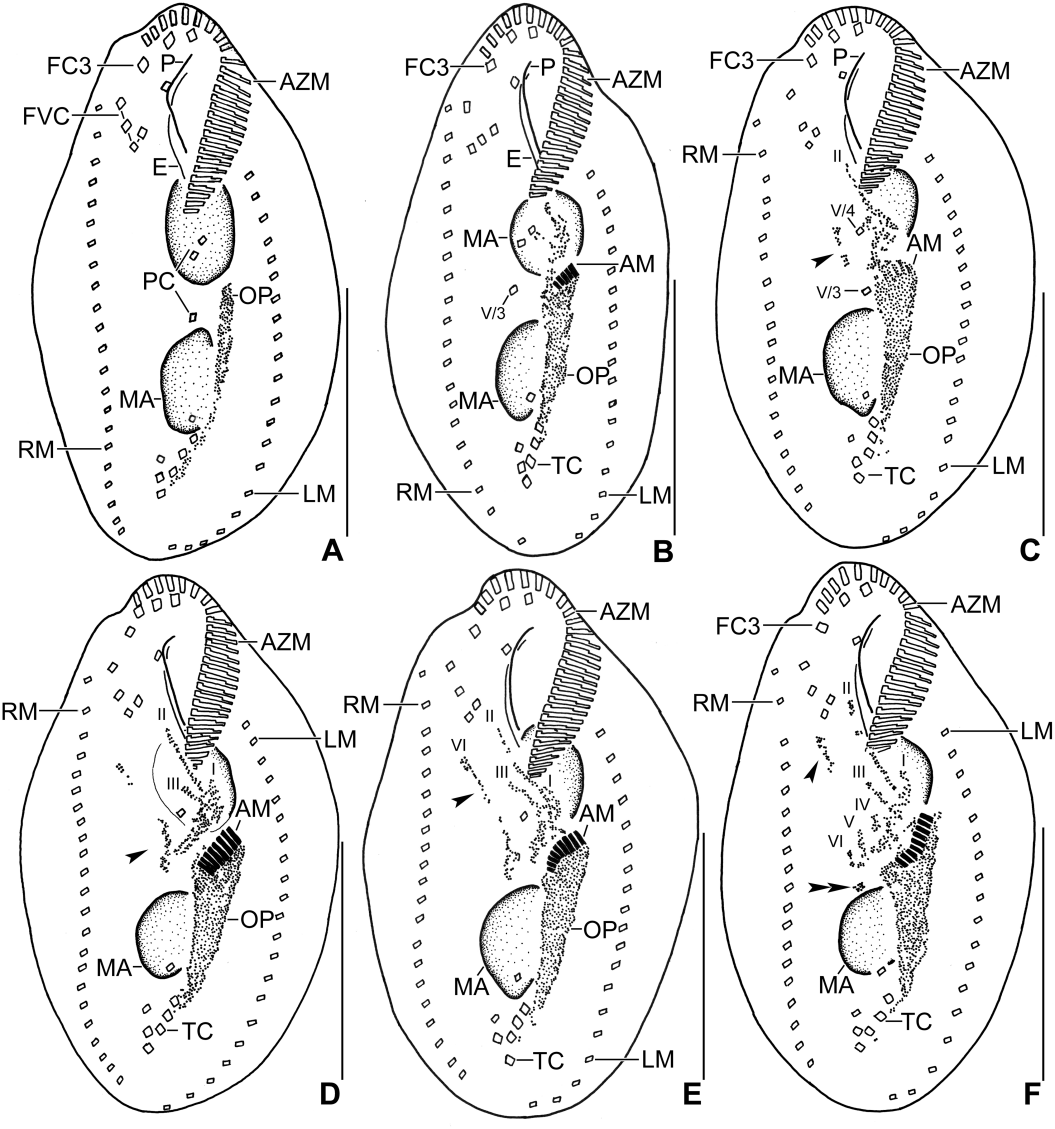
Line diagrams of protargol stained early dividers of *Metasterkiella koreana*. **(A)** Oral primordium develops close to transverse cirri. **(B)** Two anlagen arise from anterior of the oral primordium. (**C, D**) Anlage II of the opisthe moves anteriorly incorporating the postoral cirrus IV/2, part of this anlage merges with the parental buccal cirrus in early divider. Arrowhead points to the de novo origin of the rightmost anlagen for the proter and the opisthe. (**E, F**) The anterior portion of the de novo originated anlage moves anteriorly; while the posterior portion elongates incorporating cirrus V/3 and splits longitudinally forming anlagen V and VI of the opisthe. Postoral cirrus V/4 disaggregates and contributes to form the growing anlage IV of the opisthe. A rare specimen (F), showing the late disaggregation of cirrus V/3 (double arrowheads), already dissolved in previous stages. Overall, six parental cirri (II/2, III/2, IV/2, IV/3, V/3, and V/4) disaggregate to give rise to five fronto-ventral—transverse anlagen for proter and opisthe. AM, adoral membranelles; AZM, adoral zone of membranelles; E, endoral membrane; FC3, frontal cirrus 3; FVC, frontoventral cirri; LM, left marginal row; MA, macronuclear nodules; OP, oral primordium; P, paroral membrane; PC, postoral cirri; RM, right marginal row; TC, transverse cirri; V/3, V/4, postoral ventral cirri. Numerals denote cirral anlagen. Scale bars = 40 μm.

**Fig 5:**
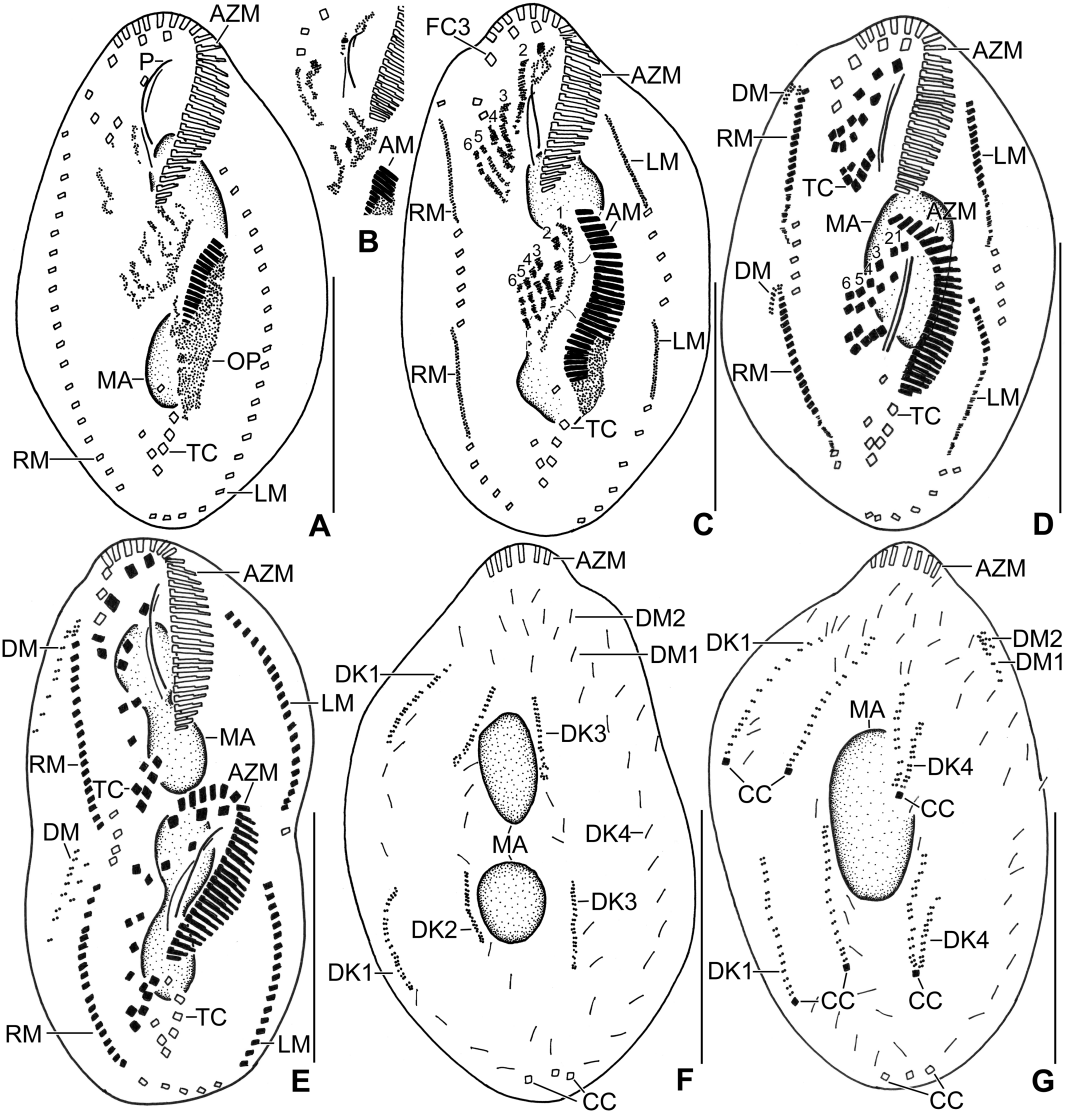
Line diagrams of protargol stained early (A–C, F), middle (D, G), and late dividers (E) of *Metasterkiella koreana*. (**A, B**) The anterior portion of the de novo originated anlage splits longitudinally forming anlagen V and VI of the proter. Cirrus IV/3 begins to disagreegate forming proter’s anlage IV, cirrus III/2 form the anlage III. **(C)** Four anlagen for marginal cirri develop incorporating four to five parental marginal cirri in the proter and the opisthe. (**D, E**) The newly formed fronto-ventral-transverse cirri migrate to their specific sites and dorsomarginal kineties develop close to the newly formed right marginal row. (**F, G**) Specimens, showing the details of the morphogenetic event on the dorsal surface with respect to the nuclear division. Within row formation of the anlagen for dorsal kineties 1–3 (F). Dorsal kinety 3 undergoes simple fragmentation forming kineties 3 and 4 (G). Caudal cirri are formed at the posterior end of dorsal kineties 1, 2, and 4, and the newly formed dorsomarginal kineties shift to the dorsal surface. AM, adoral membranelles; AZM, adoral zone of membranelles; CC, caudal cirri; DK1–4, dorsal kineties; DM1,2, dorsomarginal kineties; FC3, frontal cirrus 3; LM, left marginal row; MA, macronuclear nodules; OP, oral primordium; P, paroral membrane; RM, right marginal row; TC, transverse cirri; Numbers denote cirral anlagen. Scale bars = 40 μm.

**Fig 6:**
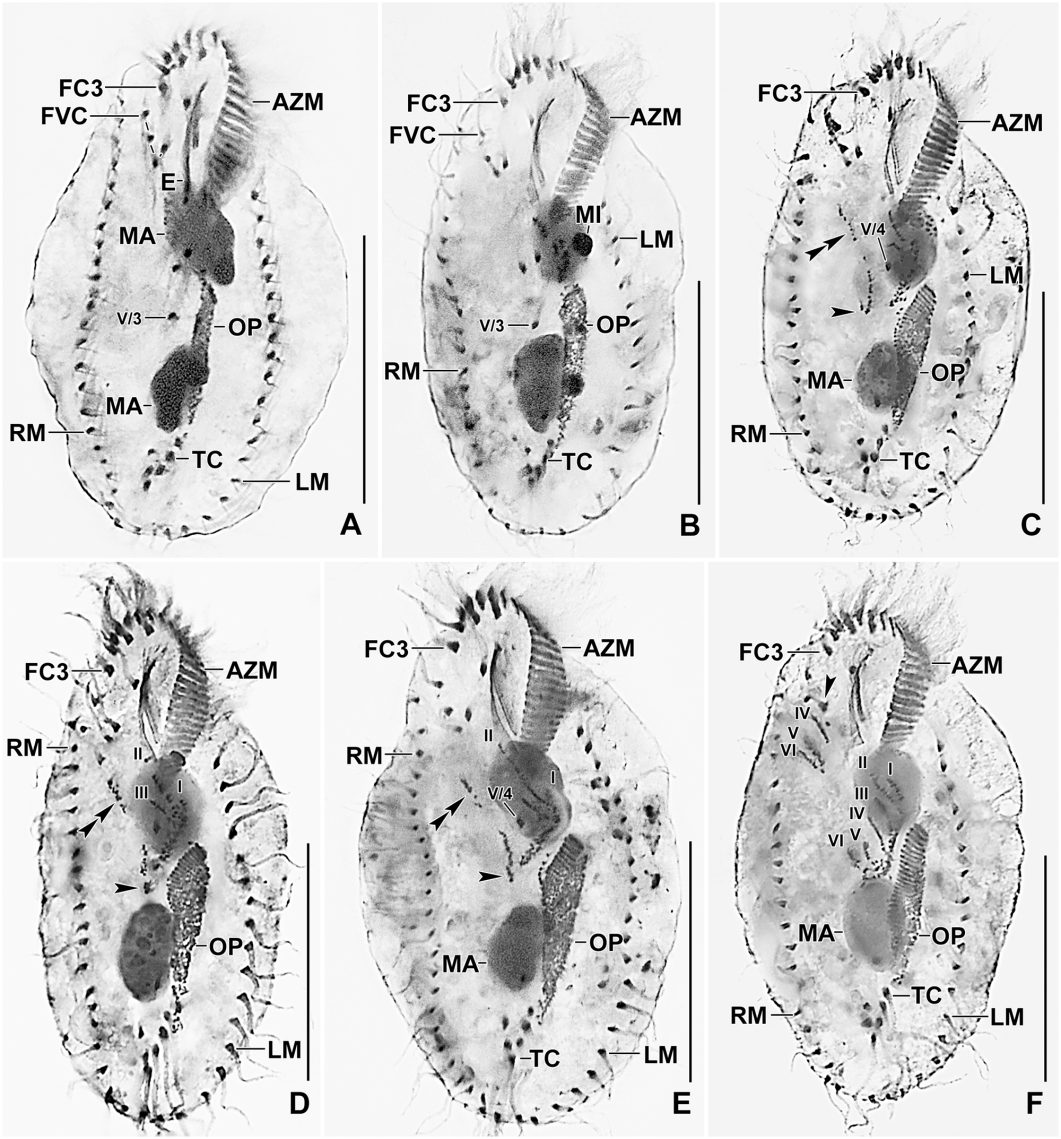
Photomicrographs of *Metasterkiella koreana* after protargol impregnation. For explanation refer to the legend of Fig 4A–4F. Double arrowheads and arrowheads in (C–E) point the anterior and the posterior portion of the rightmost anlagen for the proter and the opisthe which originates de novo. Arrowhead in (F) points to the disaggregating cirrus III/2 which forms anlage III of the proter. AZM, adoral zone of membranelles; E, endoral membrane; FC3, frontal cirrus 3; FVC, frontoventral cirri; LM, left marginal row; MA, macronuclear nodules; MI, micronuclei; OP, oral primordium; RM, right marginal row; TC, transverse cirri; V/3, V/4, postoral ventral cirri. Numerals denote cirral anlagen. Scale bars = 40 μm.

**Fig 7:**
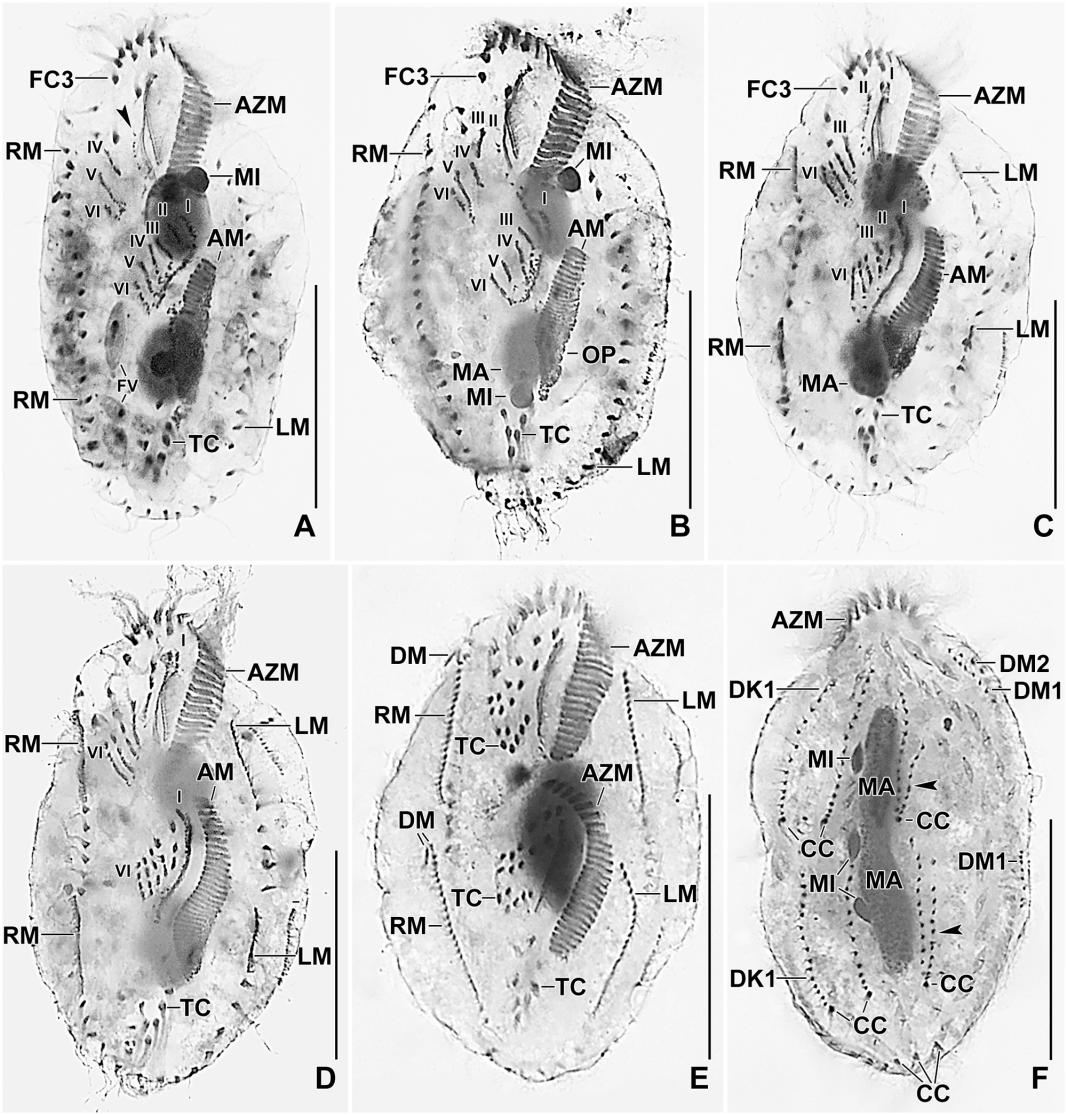
Photomicrographs of *Metasterkiella koreana* after protargol impregnation. For explanation refer to the legend of Fig 5A–5G. Arrowhead in (A) points to the anterior portion of the opisthe’s anlage II which moves anteriorly of the buccal vertex and merges with the parental buccal cirrus in early dividers. Arrowheads in (F) point to dorsal kinety 4 for the proter and the opisthe, which is formed by the simple fragmentation of dorsal kinety 3. AM, adoral membranelles; AZM, adoral zone of membranelles; CC, caudal cirri; DK1, dorsal kinety 1; DM1,2, dorsomarginal kineties; FC3, frontal cirrus 3; FV, food vacuole; LM, left marginal row; MA, macronuclear nodules; MI, micronuclei; OP, oral primordium; RM, right marginal row; TC, transverse cirri; Numerals denote cirral anlagen. Scale bars = 40 μm.

The ontogenetic stages show some variability, however, two distinguishing features were recorded, i.e., involvement of cirrus V/3 in anlagen formation and a common origin of anlagen II, V, and VI for the proter and the opisthe (Figs 4A–4F, 5A–5G, 6A–6F and 7A–7F).

The oral primordium develops close to transverse cirri and extends towards the buccal vertex (Figs 4A and 6A). The scattered basal bodies at the anterior end of the oral primordium develop into the opistheʼs anlagen I and II (Figs 4B and 6B). Cirrus IV/2 disaggregates and forms the opistheʼs anlage III (Figs 4C and 6C). A group of basal bodies originates de novo on the right side of cirrus V/4 and splits transversely. The anterior portion of this group proliferates anteriorly, forming the proter’s anlagen V and VI; the posterior portion elongates posteriorly by incorporating cirrus V/3, which further splits longitudinally to form the opistheʼs anlagen V and VI (Figs 4C–4F, 5A, 5B, 6C–6F and 7A and 7B). Anlage II of the opisthe extends with anterior portion crossing the buccal vertex and joining the disaggregating buccal cirrus in early dividers (Figs 4C–4F and 6C–6F). The disaggregation of cirrus V/4 forms the opisthe anlage IV (Figs 4F and 6F). Anlage I of the proter, i.e., the partially reorganized paroral and endoral, generates first frontal cirrus I/1 as well as the paroral and the endoral for the proter (Figs 5B–5D and 7C and 7D). Cirri III/2 and IV/3 disaggregate and give rise to the anlagen III and IV of the proter (Figs 4A, 4B and 7A and 7B). In the opisthe, anlage I separates from the posterior ends of anlagen II to VI and forms the paroral, endoral and cirrus I/1 (Figs 5C and 7A). Thus, six parental cirri and parental undulating membranes are involved in anlagen formation. The 18 frontal—ventral—transverse cirri arise from these anlagen, splitting in a 1, 3, 3, 3, 4, 4 pattern (Figs 5D, 5E and 7D and 7E). A new adoral zone of membranelles for the opisthe develops from the oral primordium, while the parental adoral zone of membranelles is retained unchanged for the proter.

The marginal anlagen arise at each of two levels by “within-row” anlagen formation utilizing one or two of the parental cirri at each level. The marginal anlagen elongate deploying four or five parental cirri and differentiate into new marginal rows. The remaining parental marginal cirri are resorbed (Figs 5C–5E and 7C–7E).

On the dorsal surface, three anlagen are formed within row from dorsal kineties 1, 2 and 3 at two levels (one set for the proter and one for the opisthe) (Figs 5F, 5G and 7F). The third dorsal primordium fragments at the middle giving rise to the third and fourth kineties. The two dorso-marginal rows arise near the right marginal row (Figs 5D, 5E and 7E). Each caudal cirrus originates at the posterior end of the new dorsal kineties 1, 2, and 4 (Figs 5G and 7F).

Nuclear division proceeds in the usual manner for oxytrichids. In middle dividers the macronuclear nodules fuse to form a single mass which divides twice to produce the typical four nodules in late dividers (Figs 5C–5G and 7A–7F). The micronuclei undergo mitotic division in the usual manner.

### SSU rRNA gene sequence and phylogeny

**Fig 8:**
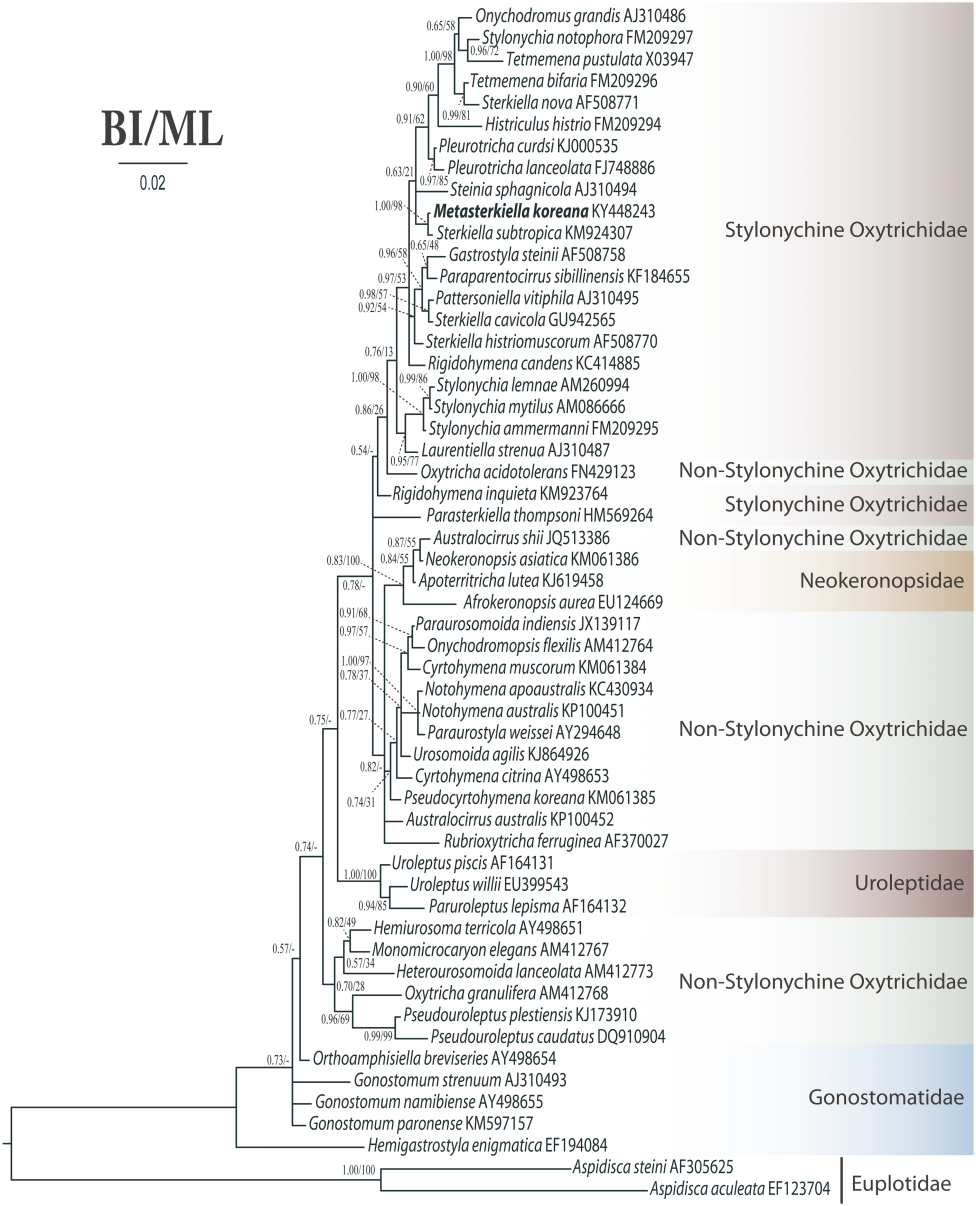
Bayesian tree inferred from the SSU rRNA gene sequences, showing the phylogenetic position of *Metasterkiella koreana* (bold). Codes following the names are GenBank accession numbers. Numbers at the nodes represent the Bayesian inference (BI) posterior probabilities and the maximum likelihood bootstrap values out of 1000 replicates. A hyphen (-) represents differences between the BI and ML tree topologies. The scale bar corresponds to two substitutions per 100 nucleotide positions.

The SSU rRNA gene sequence of *Metasterkiella koreana* is 1,652 bp in length and has a GC content of 45.03%. It has been deposited in the NCBI database under the accession number KY448243. Phylogenetic trees inferred from the SSU rRNA gene sequences using ML and BI present similar topologies; therefore, only the BI tree is shown here (Fig 8). Phylogenetic analyses consistently place the new species within the stylonychine oxytrichids, clustering in a clade with *Sterkiella subtropica*. However, the monophyly of *M*. *koreana* and *S*. *cavicola* was rejected by the AU test (p = 0.033) and the monophyly of *M*. *koreana* and all other *Sterkiella* species was refuted at p = 0.0005.

## Discussion

### Justification of the genus *Metasterkiella*

Berger and Foissner [24] and Berger [1] divided the 18-cirri oxytrichids into the Stylonychinae (rigid body, lack of cortical granules, cirrus V/3 not involved in anlagen formation) and Oxytrichinae (flexible body, cirrus V/3 involved in anlagen formation). However, a recent redescription of *Rigidohymena candens* showed that its body is flexible although being a stylonychiid ciliate [25]. Nevertheless, its stylonychiid home is supported by the ontogenetic inactivity of cirrus V/3 and by the 18S rRNA gene. This indicates that cell rigidity might not be a reliable diagnostic feature of the genus *Rigidohymena* and the subfamily Stylonychinae.

The genus *Sterkiella* possesses a semirigid body and cirrus V/3 does not participate in anlagen formation [2]. Furthermore, anlagen form separately for the proter and the opisthe [2]; except for a population of *Sterkiella histriomuscorum* described by Berger et al. [26] showing a common origin of anlagen for proter and the opisthe with intact V/3. The morphology of the present species resembles that of *Sterkiella* species, however its ontogenesis differs significantly, i.e., at least three common anlagen are formed and cirrus V/3 is involved in anlagen formation. We consider this as an important difference, especially when this feature (V/3 participation) has been used to separate species at the genus level (e.g., *Rigidohymena*, *Australocirrus*). Therefore, we propose a new genus, *Metasterkiella*, for our new species. Classification in a separate genus might help to reduce the non-monophyly problem of the genus *Sterkiella*. The population described by Berger et al. [26] shows another morphogenetic pattern, i.e., cirrus V/3 remains intact and some cirral anlagen have a common origin. Possibly, a new genus will be needed for this population. A further support for establishment of *Metasterkiella* comes from a discovery of the genus *Fragmospina* Foissner, 2016. Its type species, *F*. *depressa* Foissner, 2016, resembles *Sterkiella* species, especially *S*. *histriomuscorum* but differs in the course of the paroral membrane which almost touches frontal adoral membranelles.

### Comparison of *Metasterkiella koreana* n. gen., n. sp. with related species

In terms of the number of macronuclear nodules as well as ventral and dorsal ciliature, *Metasterkiella koreana* resembles *Sterkiella subtropica* Chen et al., 2015, *S*. *histriomuscorum* (Foissner et al., 1991) Foissner, Blatterer, Berger and Kohmann, 1991, *S*. *nova* Foissner and Berger, 1999, and *Fragmospina depressa* Foissner, 2016.

*Metasterkiella koreana* can be separated from *Sterkiella subtropica* by a combination of features, i.e., soil; 0‰ salinity (vs. marine; 29‰ salinity) habitat (Note that the specimens of *M*. *koreana* died by bursting/forming atypical structures when cultured in slightly increased salt concentration (1–3 ‰) using filtered and artificial sea water), smaller body size in vivo 75–100 × 40–75 μm (vs. 100–200 × 35–70 μm), and slightly lower number of adoral membranelles 21–28 (vs. 25–39). Minor differences include a slightly higher number of bristles in dorsal kineties, i.e., DK1 14–20 (vs. 21, data from Fig 1C of Chen et al. [27]), DK4 8–11 (vs. 13, data from Fig 1C of Chen et al. [27]), and during ontogenesis, i.e., early fragmentation (in mid divider vs. not recognizable in late divider; Fig 3h, I in Chen et al. [27]) of dorsal kinety 3. Considering these differences probably a subspecies rank would be more appropriate for the new species, as also indicated by SSU rRNA gene sequences which differ only by a single nucleotide position. However, it has been reported that SSU rRNA gene sequences are not informative for separating different species [28, 29], i.e., morphologically distinct species are shown genetically closely related by SSU rRNA gene sequences; even some species have identical SSU rRNA gene sequence but differ at other rRNA regions [29].

Most of the morphological characters of *Metasterkiella koreana* overlaps with *S*. *histriomuscorum* and *S*. *nova*, i.e., body length (65–83 vs. 65–106 and 85–150) in protargol preparation, number of adoral membranelles (21–28 vs. 28–39 and 30–39), and number of right (19–23 vs. 19–27 and 18–25) and left (18–22 vs. 16–23 and 17–23) marginal cirri [1, 30]. However, ontogenetic process of *Metasterkiella koreana* differs significantly from *S*. *histriomuscorum* and *S*. *nova*, i.e., cirrus V/3 is involved (vs. intact) during anlagen formation [1, 30].

*Fragmospina depressa* can be distinguished from this new species by having a paroral membrane close (vs. distant) to the adoral membranelles and the structure of resting cyst, i.e., spinous (vs. wrinkled) surface.

### Phylogenetic position of *Metasterkiella koreana*

Phylogenetic analyses placed *Metasterkiella koreana* close to *Sterkiella subtropica*, with full support (1.00 BI and 98% ML; Fig 8). Although these two species differ in their habitat, ciliature (minor difference), and interestingly in the ontogenetic pattern; it has been reported in Chen et al. [27] that the ontogenesis of *S*. *subtropica* is very similar to that of the type species, *Sterkiella cavicola*, in which separate anlagen are formed for the proter and the opisthe [2, 27], whereas three common anlagen are formed in the new species. The single nucleotide difference in the SSU rRNA gene of these two species indicate that *S*. *subtropica* probably belongs to *Metasterkiella*; since, both species group away from other *Sterkiella* species. A detailed reinvestigation on the ontogenesis of *Sterkiella subtropica* will confirm its final assignment; however, considering the significant difference in the ontogenesis we are confident in raising the new species up to the genus level. In support of our classification, several examples exists like, *Parasterkiella thompsoni* (Foissner, 1996) Küppers et al., 2011, *Fragmospina depressa*, which would have been easily identified as *Sterkiella* species but separated based on detailed investigations on morphology and cyst structure. At the end, it is clear that addition of related molecular sequences, e.g., *Fragmospina*, *S*. *histriomuscorum* populations, and gene sequences from other loci will further clarify whether the genus *Sterkiella* is monophyletic or not. Furthermore, it would also not be surprising to find different morphogenetic patterns in the species of the genus *Sterkiella*, e.g., *S*. *histriomuscorum* complex, *S*. *tricirrata*, which are often considered as synonyms.

### Ciliates from petroleum contaminated soils

To the best of our knowledge, this is the first report on the description of a novel ciliate species from petroleum contaminated soil. However, some reports exist on the response of certain ciliates species to crude oil contamination [31, 32]. The contamination was a result of an accidental leakage from the industrial plant in Onsan, Ulsan, South Korea. Thereby, causing spread of crude oil in the surrounding area. When the samples were collected the soil was under treatment for recovery, however, the presence of small oil droplets were still seen in the Petri dish. Interestingly, after rewetting the soil samples very few ciliates excysted, the new species was found to be freely moving around the petroleum oil droplets in the Petri dish, indicating its probable tolerance for petroleum contamination. Whether the new species have some role in purification of petroleum oil needs further investigation at the molecular as well as biochemical level.

Phylum Ciliophora Doflein, 1901
Class Spirotrichea Bütschli, 1889
Order Sporadotrichida Fauré-Fremiet, 1961
Family Oxytrichidae Ehrenberg, 1838

## Genus *Metasterkiella* n. gen.

### Diagnosis

Body Semirigid. Frontal-ventral-transverse cirri arranged in typical oxytrichid pattern. One right and one left row of marginal cirri. Six dorsal kineties including two dorsomarginal rows, kinety 3 with simple fragmentation; caudal cirri present. Undulating membranes in *Oxytricha* pattern. Common origin of anlagen II, V and VI for proter and opisthe, cirrus V/3 involved in anlagen formation.

### Type species

*Metasterkiella koreana* n. sp.

### Species assigned

*Metasterkiella koreana* n. sp.

### Etymology

Composite of the Greek prefix *meta* (next to, among after) and the genus-group name *Sterkiella*, referring to the similarity in ciliature with *Sterkiella*.

## *Metasterkiella koreana* n. sp.

### Diagnosis

Size about 85 × 50 μm in vivo; body elongate to broadly ellipsoidal. Nuclear apparatus composed of two ellipsoidal macronuclear nodules and two micronuclei on average. Invariably, 18 fronto-ventral-transverse cirri. Right and left marginal rows composed of an average of 21 and 20 cirri, respectively. Adoral zone 39% of body length and composed of an average of 24 membranelles. Three narrowly spaced, inconspicuous caudal cirri in body’s midline. Resting cyst with wrinkled surface. Soil habitat.

### Type locality

Soil samples collected from the spilled oil treatment facility, Onsan, Ulsan, South Korea (35° 24' 56"N; 129° 20' 38"E).

### Type material

The slide containing the holotype specimen (Figs 2F and 3A) and two paratype slides with protargol-stained morphostatic specimens have been deposited at the National Institute of Biological Resources (NIBR), Incheon, Korea with registration numbers NIBRPR0000107886, NIBRPR0000107887, and NIBRPR0000107888. The slides contain many specimens, with relevant cells marked by a black ink circle on the cover glass. The SSU rRNA gene sequence is deposited in GenBank (accession number: KY448243).

### Etymology

The species-group name *koreana* refers to the country where the species was discovered, i.e., Korea.

### Occurrence and ecology

As yet found only at the type location. At the time of sampling, soil was under treatment for spilled crude oil.

## Supporting information

S1 Fig Sampling images with the Safety Manager. (PDF)

S1 File Table A: Nucleotide differences (nt) in number (lower diagonal) and sequence similarity in % (upper diagonal) based on 18S/ITS/D1D2 of 28S rRNA gene sequences. Table B: Sequence comparison based on 18S, ITS, 28S (D1D2) rRNA gene sequences. Note that, *S*. *subtropica* and *S*. *cavicola* has only 18S rRNA gene sequences available in the database.
(DOCX)

## References

[1] Berger H. Monograph of the Oxytrichidae (Ciliophora, Hypotrichia). Monogr Biol. 1999; 78: 1–1080.

[2] Foissner W, Agatha S, Berger H. Soil ciliates (Protozoa, Ciliophora) from Namibia (Southwest Africa), with emphasis on two contrasting environments, the Etosha region and the Namib desert. Part I: text and line drawings. Part II: photographs. Denisia. 2002; 5: 1–1459.

[3] Foissner W. Terrestrial and semiterrestrial ciliates (Protozoa,Ciliophora) from Venezuela and Galápagos. Denisia. 2016; 35: 1–912.

[4] Kumar S, Foissner W. High cryptic soil ciliate (Ciliophora, Hypotrichida) diversity in Australia. Eur J Protistol. 2016; 53: 61–95. 10.1016/j.ejop.2015.10.001 26844781

[5] Kumar S, Bharti D, Quintela-Alonso P, Shin MK, La Terza A. Fine-tune investigations on three stylonychid (Ciliophora, Hypotricha) ciliates. Eur J Protistol. 2016; 56: 200–218. 10.1016/j.ejop.2016.09.006 27743538

[6] Foissner W, Xu K. Monograph of the Spathidiida (Cilio-phora, Haptoria). Vol. I: Protospathidiidae, Arcuospathidiidae, Apertospathulidae. Monogr Biol. 2007; 81: 1–485.

[7] Foissner W, Wolf KW, Kumar S, Xu K, Quintela-Alonso P. Five new spathidiids (Ciliophora: Haptoria) from Caribbean tank bromeliads. Acta Protozool. 2014; 53: 159–194.

[8] Blatterer H, Foissner W. Morphological and ontogenetic comparison of two populations of *Parentocirrus hortualis* Voss 1997 (Ciliophora, Hypotrichida). Linzer Biol Beitr. 2003; 35: 831–854.

[9] Kumar S, Kamra K, Bharti D, La Terza A, Sehgal N, Warren A, et al Morphology, morphogenesis, and molecular phylogeny of *Sterkiella tetracirrata* n. sp. (Ciliophora, Oxytrichidae), from the Silent Valley National Park, India. Eur J Protistol. 2015; 51: 86–97. 10.1016/j.ejop.2014.12.002 25625942

[10] Foissner W. Soil protozoa: fundamental problems, ecological significance, adaptations in ciliates and testaceans, bioindicators, and guide to the literature. Progr Protistol. 1987; 2: 69–212.

[11] Kamra K, Sapra GR. Partial retention of parental ciliature during morphogenesis of the ciliate *Coniculostomum monilata* (Dragesco and Njiné, 1971) Njiné, 1978 (Oxytrichidae, Hypotrichida). Eur J Protistol. 1990; 25: 264–278. 10.1016/S0932-4739(11)80179-3 23195974

[12] Wallengren H. Zur Kenntnis der vergleichenden Morphologie der hypotrichen Infusorien. Bih K svensk VetenskAkad Handl. 1900; 26: 1–31.

[13] Gong J, Kim S-J, Kim S-Y, Min G-S, Roberts DMcL, Warren A, et al Taxonomic redescriptions of two ciliates, *Protogastrostyla pulchra* n. g., n. comb. and *Hemigastrostyla enigmatica* (Ciliophora: Spirotrichea: Stichotrichia), with phylogenetic analyses based on 18S and 28S rRNA gene sequences. J Eukaryot Microbiol. 2007; 54: 468–478. 10.1111/j.1550-7408.2007.00288.x 18070324

[14] Medlin L, Elwood HJ, Stickel S, Sogin ML. The characterization of enzymatically amplified eukaryotic 16S-like rRNA coding regions. Gene. 1988; 71: 491–499. 3224833 10.1016/0378-1119(88)90066-2

[15] Katoh K, Standley DM. MAFFT multiple sequence alignment software version 7: improvements in performance and usability. Mol Biol Evol. 2013; 30: 772–780. 10.1093/molbev/mst010 23329690 PMC3603318

[16] Castresana J. Selection of conserved blocks from multiple alignments for their use in phylogenetic analysis. Mol Biol Evol. 2000; 17: 540–552. 10742046 10.1093/oxfordjournals.molbev.a026334

[17] Ronquist F, Teslenko M, van der Mark P, Ayres DL, Darling A, Hohna S, et al MrBayes 3.2: efficient Bayesian phylogenetic inference and model choice across a large model space. Syst Biol. 2012; 61: 539–542. 10.1093/sysbio/sys029 22357727 PMC3329765

[18] Posada D. jModelTest: Phylogenetic Model Averaging. Mol Biol Evol. 2008; 25: 1253–1256. 10.1093/molbev/msn083 18397919

[19] Stamatakis A. RAxML version 8: a tool for phylogenetic analysis and post-analysis of large phylogenies. Bioinformatics. 2014; 30: 1312–1313. 10.1093/bioinformatics/btu033 24451623 PMC3998144

[20] Miller MA, Pfeiffer W, Schwartz T. Creating the CIPRES Science Gateway for inference of large phylogenetic trees. In: Proceedings of the Gateway Computing Environments Workshop (GCE), 14 November 2010. New Orleans, LA. p. 1–8; 2010.

[21] Shimodaira H. An approximately unbiased test of phylogenetic tree selection. Syst Biol. 2002; 51: 492–508. 10.1080/10635150290069913 12079646

[22] Shimodaira H, Hasegawa M. CONSEL: for assessing the confidence of phylogenetic tree selection. Bioinformatics. 2001; 17: 1246–1247. 11751242 10.1093/bioinformatics/17.12.1246

[23] Shazib SUA, Vďačný P, Kim JH, Jang SW, Shin MK. Molecular phylogeny and species delimitation within the ciliate genus *Spirostomum* (Ciliophora, Postciliodesmatophora, Heterotrichea), using the internal transcribed spacer region. Mol Phylogenet Evol. 2016; 102: 128–144. 10.1016/j.ympev.2016.05.041 27261253

[24] Berger H, Foissner W. Cladistic relationships and generic characterization of oxytrichid hypotrichs (Protozoa, Ciliophora). Arch Protistenkd. 1997; 148: 125–155.

[25] Chen X, Yan Y, Hu X, Zhu M, Ma H, Warren A. Morphology and morphogenesis of a soil ciliate, *Rigidohymena candens* (Kahl, 1932) Berger, 2011 (Ciliophora, Hypotricha, Oxytrichidae), with notes on its molecular phylogeny based on small-subunit rDNA sequence data. Int J Syst Evol Microbiol. 2013; 63: 1912–1921. 10.1099/ijs.0.048611-0 23456808

[26] Berger H, Foissner W, Adam H. *Morphological* variation and comparative analysis of morphogenesis in *Parakahliella macrostoma* (Foissner, 1982) nov. gen. and *Histriculus muscorum* (Kahl, 1932), (Ciliophora, Hypotrichida). Protistologica. 1985; 21: 295–311.

[27] Chen X, Gao F, Al-Farraj S, Al-Rasheid K, Xu K, Song W. Morphology and morphogenesis of a novel mangrove ciliate, *Sterkiella subtropica* sp. nov. (Protozoa, Ciliophora, Hypotrichia), with phylogenetic analyses based on small-subunit rDNA sequence data. Int J Syst Evol Microbiol. 2015; 65: 2292–2303. 10.1099/ijs.0.000253 25872955

[28] Pan H, Gao F, Lin X, Warren A, Song W. Three new *Loxophyllum* species (Ciliophora: Pleurostomatida) from China with a brief review of the marine and brackish *Loxophyllum* species. J Eukaryot Microbiol. 2013; 60:44–56. 10.1111/jeu.12005 23194299

[29] Santoferrara LF, McManus GB, Alder VA. Utility of genetic markers and morphology for species discrimination within the Order Tintinnida (Ciliophora, Spirotrichea). Protist. 2013; 164:24–36. 10.1016/j.protis.2011.12.002 22264493

[30] Foissner W, Berger H. Identification and ontogenesis of the nomen nudum hypotrichs (Protozoa: Ciliophora) *Oxytricha nova* (= *Sterkiella nova* sp. n.) and *O*. *trifallax* (= *S*. *histriomuscorum*). Acta Protozool. 1999; 38: 215–248.

[31] Rogerson A, Berger J. Effect of crude oil and petroleum-degrading micro-organism on the growth of freshwater and soil protozoa. J Gen Microbiol. 1981; 124: 53–59.

[32] Rogerson A, Berger J. Ultrastructural modification of the ciliate protozoan, *Colpidium colpoda*, following chronic exposure to partially degraded crude oil. Trans Am Microsc Soc. 1982; 101: 27–35.

