# Supplementary material for: Discovery of a new hypotrich ciliate from petroleum contaminated soil
Source: PLoS One. 2017 Jun 1;12(6):e0178657. doi: 10.1371/journal.pone.0178657 (PMC5453568; doi:10.1371/journal.pone.0178657)
Supplement: S1 Fig — (PDF) [file pone.0178657.s001.pdf]

Sampling from the spilled oil treatment facility, Onsan, Ulsan, South Korea, with prior permission from the Company, collection of soil samples were performed under the control of Safety Manager and the technical staff

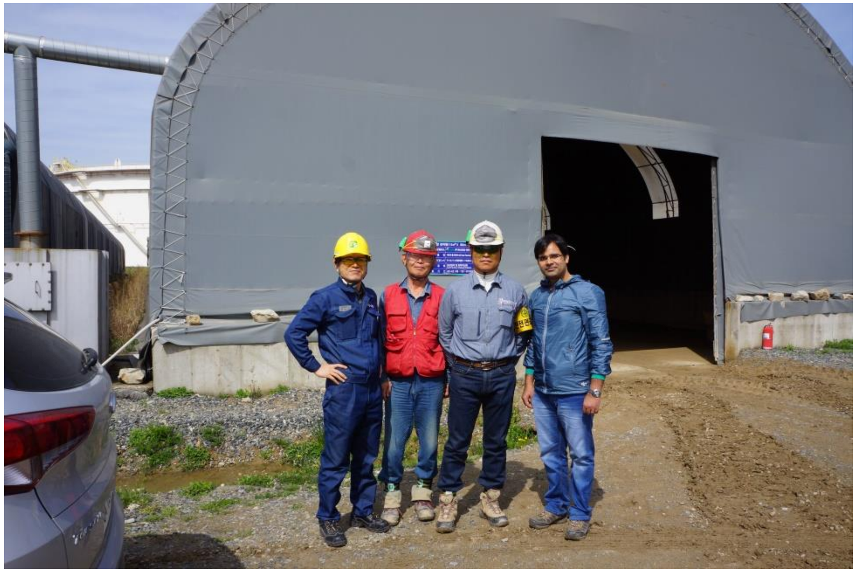

From the right: Safety Manager, two technical staff in the middle, on the left Dr. Santosh Kumar

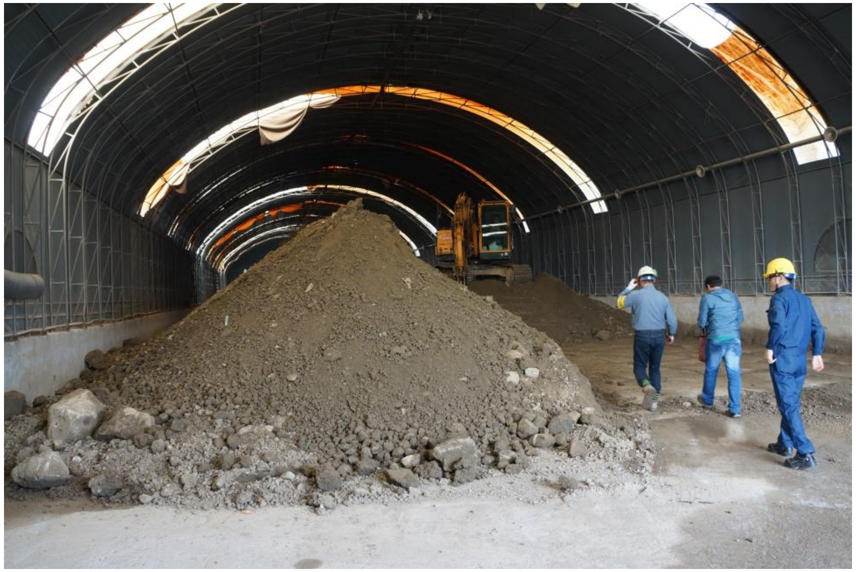

On the way to soil collection

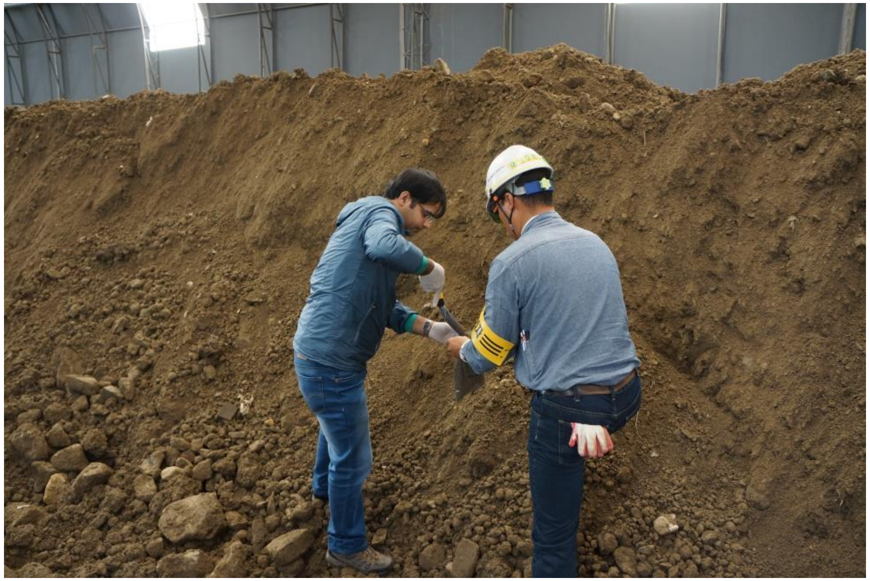

soil collection with the help of technical staff under the control of Safety Manager
