# Supplementary material for: Discovery of a new hypotrich ciliate from petroleum contaminated soil
Source: PLoS One. 2017 Jun 1;12(6):e0178657. doi: 10.1371/journal.pone.0178657 (PMC5453568; doi:10.1371/journal.pone.0178657)
Supplement: S1 File — Table A: Nucleotide differences (nt) in number (lower diagonal) and sequence similarity in % (upper diagonal) based on 18S/ITS/D1D2 of 28S rRNA gene sequences. Table B: Sequence comparison based on 18S, ITS, 28S (D1D2) rRNA gene sequences. Note that, S. subtropica and S. cavicola has only 18S rRNA gene sequences available in the database. (DOCX) [file pone.0178657.s002.docx]

**Table A:** Nucleotide differences (nt) in number (lower diagonal) and sequence similarity in % (upper diagonal) based on 18S/ITS/D1D2 of 28S rRNA gene sequences.

| Taxa | 1. | 2. | 3. |
| --- | --- | --- | --- |
| 1. *M. koreana* | --- | 98.78/93.55/96.94 | 98.10/96.22/96.54 |
| 2. *S. histriomuscorum* | 20/33/27 | --- | 98.10/93.25/96.25 |
| 3. *S. nova* | 31/19/30 | 31/35/33 | --- |

**Table B:** Sequence comparison based on 18S, ITS, 28S (D1D2) rRNA gene sequences. Note that, *S. subtropica* and *S. cavicola* has only 18S rRNA gene sequences available in the database.

| Taxa | 1. *M. koreana* | 2. *S. subtropica* | 3. *S. histriomuscorum* | 4. *S. nova* | 5. *S. cavicola* |
| --- | --- | --- | --- | --- | --- |
| 1. *M. koreana* | --- | 99.94/-/- | 98.78/93.55/96.94 | 98.10/96.22/96.54 | 98.71/-/- |
| 2. *S. subtropica* | 1/-/- | --- | 98.71/-/- | 98.04/-/- | 98.65/-/- |
| 3. *S. histriomuscorum* | 20/33/27 | 21/-/- | --- | 98.10/93.25/96.25 | 99.57/-/- |
| 4. *S. nova* | 31/19/30 | 32/-/- | 31/35/33 | --- | 98.04/-/- |
| 5. *S. cavicola* | 21/-/- | 22/-/- | 7/-/- | 32/-/- | --- |
